# The Newest of Our Creeds

**Published:** 1878-06

**Authors:** 


### EDITORIAL, ETC.

---

*The Newest of our Creeds*, Is by Dr. J. Foster Flagg, seconded by Prof. Henry S. Chase and Dr. S. B. Palmer, (see April and May Nos. of this Journal.) It is to the effect that plastic fillings are better than gold, because of the incompatibility of this last substance with tooth bone. Galvanic currents and abundance of other first-class reasons are adduced as the cause as to why the gold will not do what the other fillings do—preserve the teeth of our patients.

In this new world of ours we are always making discoveries which overtop all that is past, and compared with which all that has hitherto been found out is no more than as dust in the balance! Truly the facility with which some people change their opinions and creeds is only equalled by the agility with which the "hermit crab" slips out of his old shell into the new. The mighty champions of heavy cohesive foil, of crystal gold, of light cohesive foil, of mallets, &c., &c., after falling down on figurative knees and repenting of these sins and urging the necessity of doing their works over again, (in soft foil,) now come to the front once more after only a year's silence, and say, "*we have discovered!*"

In vian we point out to them that frail teeth have been kept for years with old-fashioned soft foil. In vain does the old operator exhibit teeth filled by dentists carefully forty years ago which are monuments of his patience and thoroughness, and point to teeth in the same mouth going to pieces because of defective gold fillings. In vain does he demonstrate that teeth are fractured by malleting, and that leaks occur under the unmanageable heavy foil. In vain—but why say a word? If neither separating or contour work saves teeth or arrests decay, if fillings do not stop its ravages, where is the utility of writing or working at all?

One thing all candid persons who have ever experimented with gold will acknowledge: *The best gold fillings are the most durable gold fillings.* Given a first-class gold filling and one of inferior quality, and even Dr. Flagg, seconded by Drs. Chase and Palmer will hardly say that that on which the most labor was spent is not most likely to serve the purpose for which it was inserted. If this be true—and the common sense of every man and the daily observation of every operator will confirm it,—it seems to our intelligence a sufficient answer to all this tirade for or against certain filling materials.

We have no objection to Amalgam. It is an excellent material for fillings. But we have, as a dentist, to say, that when a man stands up and utters as a truism that teeth are less discolored by it than when filled with gold, he is either wilfully misstating, or else he does not know what a good gold filling means. And we are more and more inclined to this last view of the case when we hear the ordinary talk about such work. Was it not Dr. Flagg who only a few years ago boasted of the facility with which he could fill cavities in teeth with some variety of plastic or shred gold? "It only took two minutes!" he said.

But the world has many such. Men who, with "the latest thing out," are just ready to outdo all their previous achievements, and whose wonder is that anything was ever accomplished before their notable discoveries were made. We must be patient with them. If "he that follows must be behind," it is implied that the leader goes ahead, and in this case a long way ahead indeed!

For ourselves, we have made a good many departures from what was old-time orthodoxy. But we have never been better pleased with our own work or that of predecessors or contemporaries, than when looking over some very bad cases filled with the "old fashioned Abbey's Soft Foil, No. 4." True, it sometimes fails, but we are quite sure that the conviction which is forced upon us in such cases of failure,—a conviction that faulty manipulation was the cause of the failure,—was nearer the truth than any theories of incompatibility. But how many of this day and generation know how to put in an old fashioned filling of soft foil!

